# Combined UV and Formic Acid Treatment Suppresses *Aspergillus flavus* and Aflatoxin B_1_ on Dried Red Chili Powder

**DOI:** 10.3390/foods14132194

**Published:** 2025-06-23

**Authors:** Xiaoman Chen, Gang Yang, Yi Zhang, Yaoyao Su, Jun Huang, Aijun Li, Kewei Chen, Muying Du, Zsolt Zalán, Sameh Awad, Jianquan Kan

**Affiliations:** 1 College of Food Science, Southwest University, 2 Tiansheng Road, Beibei, Chongqing 400715, China; chenxiaomman@163.com (X.C.); yanggangresearch@163.com (G.Y.); zhangyii2000@163.com (Y.Z.); suyaoyao799@163.com (Y.S.); huangjunresearch@163.com (J.H.); liaijun2021@163.com (A.L.); chenkewei@foxmail.com (K.C.); muyingdu_swu@163.com (M.D.); 2 Chinese-Hungarian Cooperative Research Centre for Food Science, Chongqing 400715, China; 3 Chongqing Key Laboratory of Speciality Food Co-Built by Sichuan and Chongqing, Chongqing 400715, China; 4 Laboratory of Quality & Safety Risk Assessment for Agro-Products on Storage and Preservation (Chongqing), Ministry of Agriculture, Chongqing 400715, China; 5 Food Science and Technology Institute, Hungarian University of Agriculture and Life Sciences, Buda Campus, Herman Ottó Str. 15, 1022 Budapest, Hungary; zalan.zsolt@eki.naik.hu; 6 Dairy Science and Technology Department, Faculty of Agriculture, Alexandria University, Alexandria 22302, Egypt; samehmawad@gmail.com

**Keywords:** dried chili, *Aspergillus flavus*, aflatoxin B_1_, combined prevention and control, formic acid, ultraviolet light

## Abstract

Aflatoxin contamination poses a significant food safety risk, particularly during the storage of dried chili peppers. This study evaluated the efficacy of formic acid treatment, ultraviolet (UV) treatment, and combined UV-formic acid treatment in both preventing and controlling *Aspergillus flavus* in dried red chili powder. Efficacy was assessed by measuring the growth diameter of *A. flavus* colonies on un-colonized and already colonized dried red chili powder. The optimal treatment conditions for the UV-formic acid combination were determined through single-factor experiments, orthogonal experiments, and quality assessment. Finally, the effects of the UV-formic acid combination on the cell membrane, antioxidant system, and energy metabolism of *A. flavus* were investigated. The results revealed that fumigation of un-colonized dried red chili powder with 5% formic acid for 24 h inhibited *A. flavus* growth by 93.29% and toxin synthesis by 99.41%. In contrast, treatment of already colonized chili powder with 10% formic acid inhibited *A. flavus* colony growth by 50%. Through a three-factor, three-level orthogonal experiment followed by quality testing, the optimal conditions were determined to be 8% formic acid concentration, a UV irradiation distance of 15 cm, and a treatment time of 75 min. This optimized combined treatment reduced the required fumigation time from 24 h to 1.25 h. This technique achieved complete suppression of aflatoxin B_1_ synthesis on un-colonized dried red chili powder. On already colonized chili powder, the mycelial growth inhibition rate was 48.05 ± 6.68%, and aflatoxin B_1_ synthesis was inhibited by 91.32 ± 3.15%. Quality assessment revealed that the UV-formic acid co-treatment parameters did not significantly affect key quality indicators including color, capsaicin content, total phenolic content (*p* > 0.05). Furthermore, UV-formic acid treatment disrupt the cell membrane structure of *A. flavus*, impairs its antioxidant and energy metabolism systems, and induces mitochondrial dysfunction. The study confirmed the synergistic antifungal effect of formic acid and UV, providing a potential industrialized solution for enhancing the safety and storage stability of dried chili products.

## 1. Introduction

*Aspergillus flavus* (*A. flavus*) is a saprophytic fungus responsible for contaminating food and agricultural products [1]. It produces aflatoxin B_1_ (AFB_1_), a potent mycotoxin posing a significant global food safety threat [2]. AFB_1_ is classified as a Group 1 human carcinogen by the International Agency for Research on Cancer (IARC) [3]. Prolonged exposure to AFB_1_ can lead to liver cancer, reproductive complications, cardiac problems, and immune suppression [4,5,6,7]. Globally, AFB_1_ contamination impacts approximately one-quarter of the world’s population, representing a major public health concern [8].

Red chili pepper (*Capsicum annuum* L.) is a widely used consumed spice rich in vitamins, capsaicin, and flavonoids [9]. To extend shelf life, peppers are typically dried to reduce the moisture content. However, environmental factors during storage promote microbial contamination, causing significant product waste [10]. AFB_1_ contamination occurs in chili peppers from nearly 70% of countries worldwide, with rates often exceeding 50%. In China—a major dried red chili producer—aflatoxin contamination remains serious, exceeding the national limit (5 μg/kg) in some regions [11]. Conventional control methods (high-temperature drying, γ-ray irradiation, chemical fumigation) face limitations including high energy consumption, flavor degradation, and residue risks, making them inadequate for meeting modern food industry demands for safe, efficient, and non-destructive preservation [12,13].

Ultraviolet (UV) technology is increasingly used for food preservation due to its rapid action, contactless nature, and absence of chemical residues [14]. Studies indicate that UV-C (254 nm) can damage microbial DNA and disrupt cell membrane structure [15]. However, its limited penetration into agricultural products like dried red chili peppers, which have deep surface folds and thicknesses, impeding complete elimination of internal contaminants [16]. Fumigation offers a convenient and effective method for controlling fungal growth and contamination, demonstrating particular efficacy against thicker food substrates [17]. Research by Mao et al. [18] and Bozidar et al. [19] showed UV irradiation degrades aflatoxins and inhibits *A. flavus* in peanut oil, maize, and peanuts. Formic acid, a common fumigant reagent, exerts broad-spectrum antifungal effect by acidifying the environment, disrupting cell membranes permeability, and inhibiting enzyme activity [20]. Its high volatility minimizes residual effects on product quality. Lee et al. [21] demonstrated the inactivation of *A. flavus* on raw coffee beans using organic acid vapors, including formic acid. No mycelial growth occurred on beans treated with formic acid vapor during storage, confirming its efficacy against *A. flavus*. There have been no studies on the use of formic acid to control *A. flavus* contamination on dried red chili powder, nor have formic acid and UV been used in a combined treatment.

Synergistic UV treatment combined with formic acid fumigation effectively achieves deep-level fungal suppression and extends storage duration by preventing and controlling *A. flavus* contamination in dried chili powder. This approach combines UV exposure with formic acid fumigation, potentially increasing spore sensitivity to formic acid while formic acid may potentiate UV-induced cellular damage. The combination demonstrates a synergistic antifungal effect. The combination of UV with caffeic acid significantly inhibited the growth of *E. coli* O157:H7, *Salmonella typhimurium* and *Listeria monocytogenes* in a study by Park et al. [22]. Recent studies have shown that significant synergistic inhibition can be achieved by combining physicochemical inhibition techniques in the food systems [23,24,25,26], though their application for chili pepper products remains unexplored.

This study investigates the combined inhibitory effects of UV light and formic acid fumigation on *A. flavus* growth and AFB_1_ synthesis. Orthogonal experiments were conducted to determine the optimal treatment parameters for the UV-formic acid combination, including UV irradiation distance, formic acid concentration, and treatment time. Color, capsaicin content, total phenol content (TPC), and 2,2-Diphenyl-1-picrylhydrazyl (DPPH) radical scavenging rate served as indicators for both safety and quality evaluation. The impact of the technology on the cell membrane, antioxidant capacity, and cellular energy metabolism of *A. flavus* were also investigated to verify the feasibility of the technology. This is the first application of UV and formic acid fumigation for aflatoxin contamination on dried red chili powder, providing a novel approach for the storage and preservation of agricultural products.

## 2. Materials and Methods

### 2.1. Materials and Strain Culture

*A. flavus* (CICC 2219) was purchased from the China Centre of Industrial Culture Collection (Shanghai, China). The strain was activated twice on potato dextrose agar (PDA) and kept on the slant of PDA medium at 4 °C for future use. Dried red chili peppers were obtained from a local supermarket (Chongqing, China), crushed, sieved, vacuum-sealed, sterilized, and stored at 4 °C. Trifluoroacetic acid, fluorescein diethyl acetate (FDA), hexane, acetonitrile, and methanol (HPLC grade) were purchased from Shanghai Maclin Company, China (Shanghai, China). Propidium iodide (PI), purchased from Beijing Regen Biotechnology Co (Beijing, China). All other reagents were of analytical grade.

Following the method described by Fan et al. [27], the *A. flavus* strain was inoculated on PDA and incubated at 28 °C for 5–7 d. Once the plate was covered with yellowish-green mycelium, the surface was rinsed with 1‰ Tween 80 (*w*/*w*) buffer in a sterile environment and then vigorously shaken to obtain a spore suspension. The suspension was filtered through a sterilized mycelial filter and four layers of sterile gauze. The spores were counted using a hemocytometer and diluted with sterilized 1‰ Tween-80 (*w*/*w*) buffer to the desired concentration for further use.

### 2.2. Formic Acid and UV Treatment

#### 2.2.1. Formic Acid

The fumigation method described by Li et al. was referenced and adaptively adjusted [28]. 20 mL of formic acid solution (volume fraction concentration, 1.25%, 2.5%, 5%, 10%, 15%, and 20%) was pipetted into the bottom of a 250 mL beaker. A spring was placed inside the beaker, a 60 mm Petri dish containing the samples was positioned on top of the spring. The beaker was sealed with plastic film and fumigated at room temperature for 24 h.

#### 2.2.2. Ultraviolet

According to the protocol described by Chen et al. [16], a 100 W UV lamp (254 nm) was mounted in a sealed chamber. The irradiation distance was fixed at 15 cm, with varying exposure times (0.5, 1, 1.5, 2, 2.5 h). The samples were placed under the UV lamp at an adjustable distance. Prior to treatment, the UV lamp was preheated for 30 min to ensure sterile conditions.

### 2.3. Determination of the Inhibitory Effect of Formic Acid on the Growth and AFB_1_ Synthesis of A. flavus on Dried Red Chili Powder

#### 2.3.1. Determination of the Inhibition Rate of A. flavus Colonies

To assess the inhibition of *A. flavus* on dried red chili powder using fumigation, 2.0 g of chili powder was spread in a 60 mm Petri dish, followed by inoculation of 100 μL *A. flavus* spore suspension (10^6^ CFU/mL) at the center. Based on previous experiments, formic acid fumigation concentrations were set at 1.25%, 2.5%, 5%, 10%, 15%, and 20%. Two test groups were established: one group underwent direct formic acid fumigation for 24 h, while the other group was fumigated after mycelial growth reached 10–15 mm. All samples were incubated at 33 °C for 5 d, and the colony diameters were measured daily to calculate *A. flavus* growth inhibition rates (calculation formula is shown in (1)). Triplicate samples were analyzed for AFB_1_ content.

(1) Inhibition rate%=C1−C0/C0×100%,

where *C*0 and *C*1 are the colony diameters of the control and treatment groups, respectively.

#### 2.3.2. Determination of AFB_1_ Content

AFB_1_ content was determined according to Chinese National Standard GB 5009.22-2016 [29] method II using high-performance liquid chromatography (HPLC) with pre-column derivatization. After blow-drying the immunoaffinity column with nitrogen, trifluoroacetic acid and n-hexane were added for derivatization [30]. The samples were incubated at 45 °C for 15 min in the dark, reconstituted with the initial mobile phase by under nitrogen flow, and filtered through a 0.22 μm organic membrane before HPLC fluorescence detector (FLD). The analysis of AFB_1_ was performed on a C18 column (150 mm in length, 4.6 mm in inner diameter, 5.0 μm in packing size) with a mobile phase of 55% water and 45% organic (acetonitrile: methanol = 1:1 (*v*/*v*)) at a flow rate of 1.0 mL/min, a column temperature of 40 °C, with a sample volume of 50 μL. The excitation wavelengths and emission wavelengths of the FLD were 360 and 440 nm.

### 2.4. Determination of the UV-Formic Acid Combination Approach

Based on the results of the previous experiments, a 100 W (254 nm) UV lamp and 10% formic acid was selected. With the UV irradiation distance fixed at 15 cm, different combination methods such as single, series and parallel were evaluated for their effects on *A. flavus* in dried red chili powder (Table 1). Specifically, 2.0 g samples spread in 60 mm Petri dishes underwent 90 min treatments. Single-treatment (W3) samples were fumigated with 20 mL of 10% formic acid for 90 min. Series treatments (W5) were treated with UV for 45 min, followed by fumigation with formic acid for 45 min. Parallel (W10), UV, and formic acid treatments were performed for 90 min together. All treatments were performed in triplicates.

### 2.5. Determination of the Combined Single Factor of Formic Acid and UV

After determining the treatments, the effects of three factors, namely UV irradiation distance (11, 13, 15, 17, 18.5 cm), formic acid treatment concentration (4, 6, 8, 10, 12%), and total treatment time (30, 45, 60, 75, and 90 min) on *A. flavus* growth in dried red chili powder. The orthogonal factor level matrix was established, with three biological replicates per treatment group.

### 2.6. Determination of Growth Inhibition and AFB_1_ Synthesis of A. flavus by Combined UV Formic Acid Orthogonal Test

Based on the above single-factor test, an orthogonal experimental design was implemented using the L9 (3^3^) orthogonal array to optimize three key factors: UV irradiation distance, formic acid concentration, and total treatment time. This approach aimed to determine the optimal conditions for inhibiting both growth and toxigenicity of *A. flavus* in dried red chili powder. The corresponding factors and their levels are detailed in Table 2.

### 2.7. Determination of the Quality of Dried Red Chili Powder After UV-Formic Acid Treatment

The dried red chili powder samples underwent orthogonal experimental treatments, with subsequent analysis of quality parameters. Untreated samples served as controls for comparative assessment.

#### 2.7.1. Color, Natural Coloring Matter

The brightness (*L*), red–green (*a*), and yellow–blue (*b*) values for each chili powder treatment group were measured using a colorimeter. Natural coloring matter was extracted with acetone solution, and the absorption value was measured at 460 nm according to the American Spice Trade Association [31].

#### 2.7.2. Capsaicin Content Analysis

Chromatographic analysis was performed in accordance with Chinese National Standard GB/T 21266-2007 [32] using the following conditions: Zorbax SB-C18 column (250 mm length × 4.6 mm inner diameter, 5 μm particle size), mobile phase composed of water-methanol (35:65, *v*/*v*), flow rate 1.0 mL/min, column temperature 40 °C, injection volume 10 μL, and UV detection at 280 nm.

#### 2.7.3. Total Phenolic Content (TPC)

TPC was determined by the Folin–Ciocalteu method. A 2.0 g sample was extracted with 20 mL of 80% aqueous methanol [33], ultrasonicated for 20 min, and centrifuged at 4000 rpm for 10 min. The supernatant was diluted to 50 mL. Following a modified method [34], 0.2 mL of the diluted extract was mixed with 0.5 mL Folin reagent, vortexed for 10 s, and allowed to stand for 5 min. Subsequently, 1.5 mL of 10% (*w*/*v*) sodium carbonate solution was added, and the mixture was incubated in the dark for 30 min. Absorbance was measured at 765 nm. TPC was calculated by

(2) $$X=\left(\rho \times V\right)/(m\times (100-X_{w})\times 100),$$

where X represents the TPC (in terms of gallic acid, mg/g), ρ is the mass concentration of TPC in the sample solution obtained from the standard curve (mg/mL), V represents the volume of sample solution (mL); m is the mass of the sample (g), and X_w_ is the moisture content (g/100 g).

#### 2.7.4. DPPH Radical Scavenging Rate

DPPH radical scavenging activity was determined using methanol-extracted supernatant [35]. Specifically, 0.5 mL of extract was mixed with 1 mL of DPPH methanol solution (250 μmol/L). The reaction mixture was incubated in the dark for 10 min, and absorbance was measured at 517 nm. The scavenging rate was calculated as

(3) $$DPPH\;radical\;scavenging\;rate\%=(\left(A0-A1\right)/A0)\times 100\%,$$

where A0 and A1 are the absorbance of the blank and sample, respectively.

### 2.8. Assessing the Impact of UV-Formic Acid Treatment on A. flavus Membrane Integrity

#### 2.8.1. PI and FDA Staining

The integrity of *A. flavus* spore cell membranes was assessed using FDA and PI according to Wang et al. [36]. Based on prior experimental results, UV-formic acid treatment dose was obtained. *A. flavus* spore suspension (10^6^ CFU/mL) was treated using UV-formic acid treatment (T1: formic acid fumigation concentration of 8%, UV irradiation distance of 15 cm, total treatment time of 75 min). After the treatment was completed, it was washed three times repeatedly using phosphate-buffer solution (PBS). The spore suspension was then washed twice with PBS, centrifuged and resuspended in PBS after dark treatment with FDA staining for 30 min at 37 °C and PI staining for 20 min. Finally, the spores were observed by orthogonal fluorescence microscopy.

#### 2.8.2. Leakage of Intracellular Proteins and Nucleic Acids

*A. flavus* spore suspensions underwent two UV-formic acid treatments (T1, T2: formic acid fumigation concentration of 20%, UV irradiation distance of 7.5 cm, and total treatment time of 3 h). The treated samples were processed according to Li et al. [37]: spore suspensions were centrifuged at 10,000 r/min for 10 min. Supernatant absorbances at 260 nm and 280 nm were determined by UV spectrophotometer.

#### 2.8.3. Observation of Micro-Morphology of A. flavus

Scanning electron microscopy (SEM) was employed to compare the colony morphology of *A. flavus* mycelium between UV-formic acid treated (T1) and untreated control groups. Following a modified protocol based on Li et al. [38], samples were fixed with 2.5% glutaraldehyde at 4 °C overnight, rinsed thrice with phosphate buffer, and post-fixed with 1% osmium tetroxide for 2 h. Subsequent processing included ethanol dehydration through a gradient series (30, 50, 70, 80, 90, 100%; 15 min per concentration), critical-point drying, and gold sputter coating prior to SEM observation.

### 2.9. Evaluating the Impact of UV-Formic Acid on A. flavus Antioxidant Response

Malondialdehyde (MDA) content was measured to assess lipid peroxidation levels in mycelial cell membrane, following the method of Liao et al. [39]. Briefly, 0.5 g of mycelium per group was homogenized with 3 mL of 10% (*w*/*v*) trichloroacetic acid (TCA) in an ice bath. The homogenate was centrifuged at 10,000× *g* for 10 min (4 °C). Subsequently, 2.0 mL of supernatant was mixed with thiobarbituric acid (TBA) reagent and incubated in a boiling water bath (100 ± 2 °C, 20 min). After cooling to room temperature, samples were re-centrifuged. The absorbance values at 450, 532, and 600 nm were determined to calculate the MDA content:

(4) $$\mathrm{MDA}\mathrm{content}\;(\mu \mathrm{mol}/g)=\frac{\left[6.45\times \left({OD}_{532}-{OD}_{600}\right)-0.56\times {OD}_{450}\right]\times V1}{\begin{array}{c} V2\times m\times 1000 \end{array}},$$

where V1 is the total volume of sample extract (mL), V2 is the sampling volume of sample extract (mL), and m is the weight of mycelium sample (g).

Mycelium (0.1 g) from each treatment group was used to determine superoxide dismutase (SOD) activity after T1 and T2 UV-formic acid treatments, utilizing a commercial SOD assay kit.

### 2.10. Assessment of Energy Metabolic Responses in A. flavus to UV-Formic Acid Treatment

Mycelia from both control and treated *A. flavus* groups were weighed (0.1 g per sample). Enzymatic activities of Na^+^/K^+^-ATP, NAD-isocitrate dehydrogenase (NAD-IDH) kit and succinate dehydrogenase (SDH) measured using commercial assay kits specific to each enzyme.

### 2.11. Experimental Data Processing and Analysis

Statistical analyses were conducted using excel 2010 and SPSS 18.0, employing the Waller–Duncan test for significance assessment. Differences were considered statistically significant at *p* < 0.05. The figures were generated using Origin 2024 and Adobe Illustrator CC 2014.

## 3. Results

### 3.1. Effect of Formic Acid on the Growth and AFB_1_ Synthesis of A. flavus on Dried Red Chili Powder

**Figure 1:**
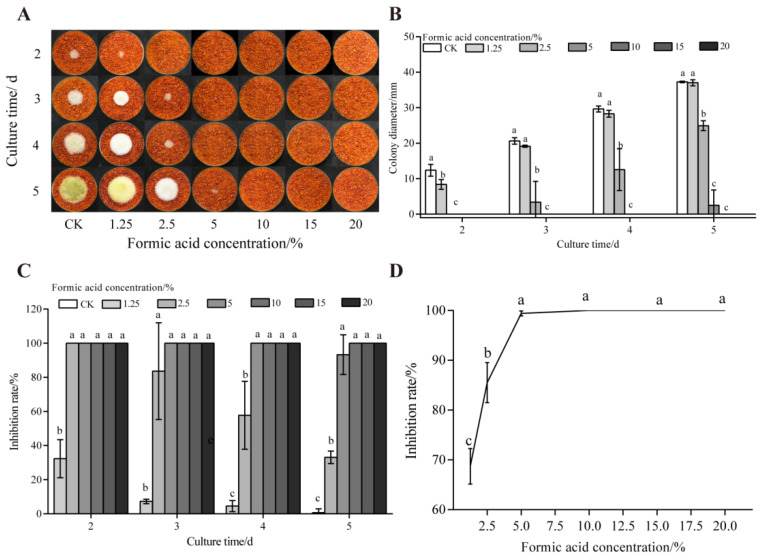
Effects of formic acid on the growth of *A. flavus* on un-colonized of dried red chili powder. (**A**) Growth of *A. flavus*; (**B**) diameter of *A. flavus*-growing colonies; (**C**) inhibition of colony growth; (**D**) inhibition of AFB_1_ synthesis. Different letters at the top of the column indicate significant differences between treatment conditions (*p* < 0.05).

As demonstrated in Figure 1, formic acid fumigation significantly inhibited *A. flavus* colony growth. No mycelial growth was observed after 2 d of treatment. On the fourth day, 2.5% formic acid treatment suppressed *A. flavus* on dried red chili powder with inhibition rates exceeding 80%. In contrast, 1.25% formic acid showed negligible inhibitory effects. Critically, higher concentrations (≥10%) completely suppressed AFB_1_ synthesis achieving 100% inhibition. This finding is consistent with previous research findings on the high inhibitory effect on aflatoxin production of up to 95% [40].

**Figure 2:**
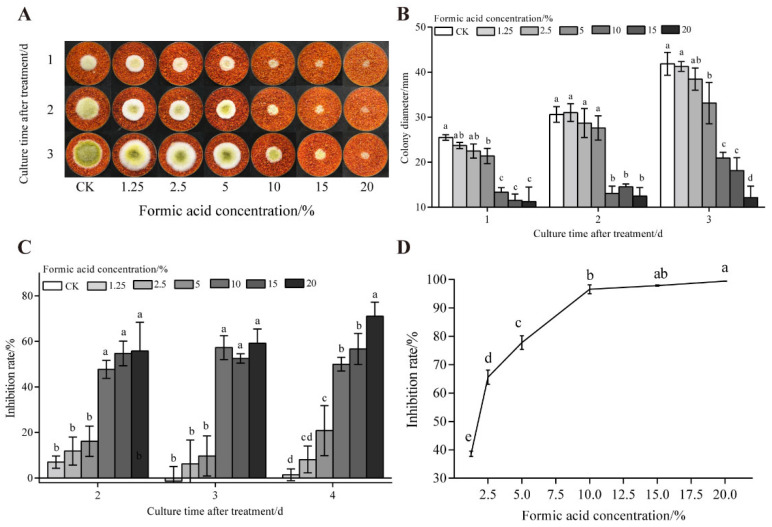
Effects of formic acid on the growth of *A. flavus* on already colonized of dried red chili powder. (**A**) Growth of *A. flavus*; (**B**) diameter of *A. flavus*-growing colonies; (**C**) inhibition of colony growth; (**D**) inhibition of AFB_1_ synthesis. Different letters at the top of the column indicate significant differences between treatment conditions (*p* < 0.05).

Figure 2 demonstrates the inhibitory effect of formic acid fumigation on already colonized chili powder. Concentrations lower than 5% showed no statistically significant suppression of fungal colonies. At ≥10% formic acid, AFB_1_ inhibition reached 97.87% (*p* < 0.05). However, prolonged fumigation with high concentrations generates volatile emissions that compromise sensory attributes due to persistent irritant odors.

### 3.2. The Effect of UV on the Growth and AFB_1_ Synthesis of A. flavus on Dried Red Chili Powder

Our prior studies [16] confirmed that UV treatment inhibits *A. flavus* growth and AFB_1_ synthesis (stronger inhibitory effects at shorter irradiation distances and longer irradiation time). Specifically, 1.5 h UV irradiation at 15 cm distance achieved 64.64% growth inhibition on already colonized chili powder. However, UV’s limited penetration depth and surface morphology interference prevent complete pathogen elimination [41]. Prolonged UV exposure may also affect chili color [42,43]. Consequently, formic acid co-treatment was investigated to enhance efficacy while reducing UV dosage.

### 3.3. The Effect of Different UV-Formic Acid Combined Approach on the Growth of A. flavus and AFB_1_ Synthesis on Dried Red Chili Powders

**Figure 3:**
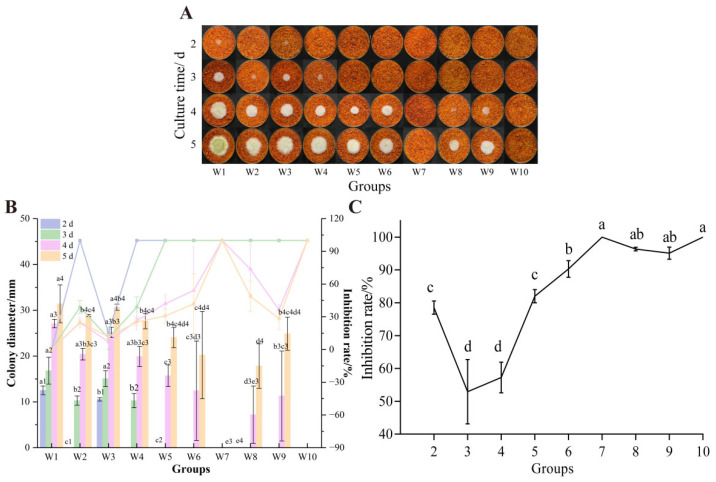
Effects of combined treatments on the growth of *A. flavus* on un-colonized dried red chili powder. (**A**) Growth of *A. flavus*; (**B**) inhibition of *A. flavus* colony diameter and colony diameter; (**C**) inhibition of AFB_1_ synthesis. Different letters at the top of the column indicate significant differences between treatment conditions (*p* < 0.05).

Optimal inhibition was achieved with 10% formic acid concentration and 15 cm UV irradiation for 1.5 h. As shown in Figure 3, among nine orthogonal treatments, W7 (UV treatment for 45 min, then formic acid and UV treatment in parallel for 45 min) and W10 (formic acid and UV treatment in parallel for 90 min) demonstrated complete suppression of *A. flavus* growth and AFB_1_ synthesis on the 5th day. This treatment is significantly better than W2 (growth inhibition rate of 25.24%, AFB_1_ synthesis inhibition rate of 78.53%) and W3 (growth inhibition rate of 11.67%, AFB_1_ synthesis inhibition rate of 52.90%). Formic acid helps in deeper penetration, while UV irradiation helps in deeper sterilization [44]. This combined treatment with UV can enhance the inhibitory effect with synergistic mechanism, which is consistent with the previous literature findings [45,46].

**Figure 4:**
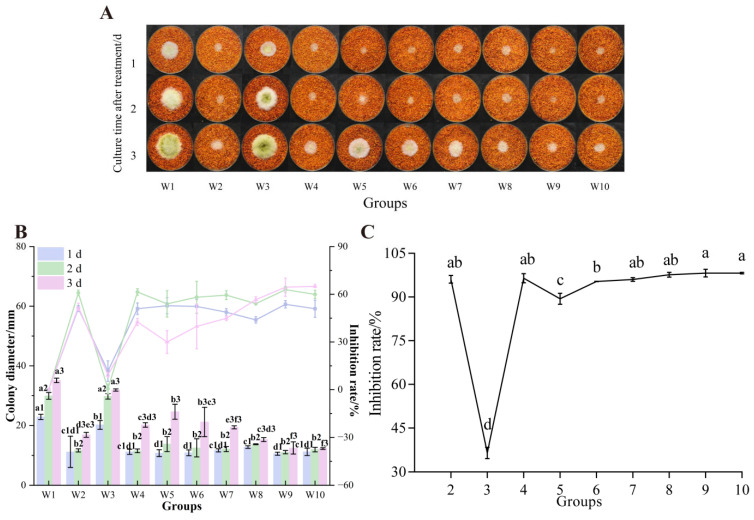
Effects of combined treatments on the growth of *A. flavus* on already colonized dried red chili powder. (**A**) Growth of *A. flavus*; (**B**) inhibition of *A. flavus* colony diameter and colony diameter; (**C**) inhibition of AFB_1_ synthesis. Different letters at the top of the column indicate significant differences between treatment conditions (*p* < 0.05).

Figure 4 illustrates the inhibitory effects on already colonized, dried red chili powder. All treatments significantly inhibited the growth of *A. flavus* colonies. Crucially, only W9 (first formic acid and UV parallel treatment for 45 min, and then UV treatment for 45 min) and W10 (formic acid and UV parallel treatment for 90 min) achieved complete suppression of colony regrowth by day 3. The W10 group exhibited the highest colony growth inhibition rate, 64.99% (Figure 4B).

These results indicate that the combined treatment outperforms single-method applications. This aligns with prior research [47] where ozone and slightly acidic electrolytic water synergism enhanced fungal inhibition in oysters. The UV-formic acid synergy achieves high-efficiency *A. flavus* control in chili powder while reducing fumigation duration. Studies have shown that organic acids can penetrate cell membranes and lower intracellular pH, leading to cytoplasmic acidification, degradation of enzymes and base analogs essential to cells, inhibition of mitochondrial respiratory chain enzyme activity (e.g., SDH), disruption of energy metabolism, and ultimately inhibition of cellular activity, leading to microbial inactivation [48]. Conversely, UV sterilization primarily induces DNA damage through thymine dimer formation, while concurrently oxidizing vital intracellular components [49]. Therefore, the combined effects of the two can simultaneously interfere with the physiological metabolism and genetic stability of *A. flavus* and block its repair mechanism, thereby enhancing antifungal efficacy. Formic acid’s high volatility and membrane permeability [50] facilitate rapid cellular internalization. Whereas UV can only achieve the effect of surface inhibition [44], their combination establishes a comprehensive antimicrobial barrier. It covers the surface of the paprika particles and internal pores, reducing the local treatment of the blind zone.

### 3.4. The Effect of Formic Acid Concentration on the Growth of A. flavus on Dried Red Chili Powder in UV-Formic Acid Treatment

**Figure 5:**
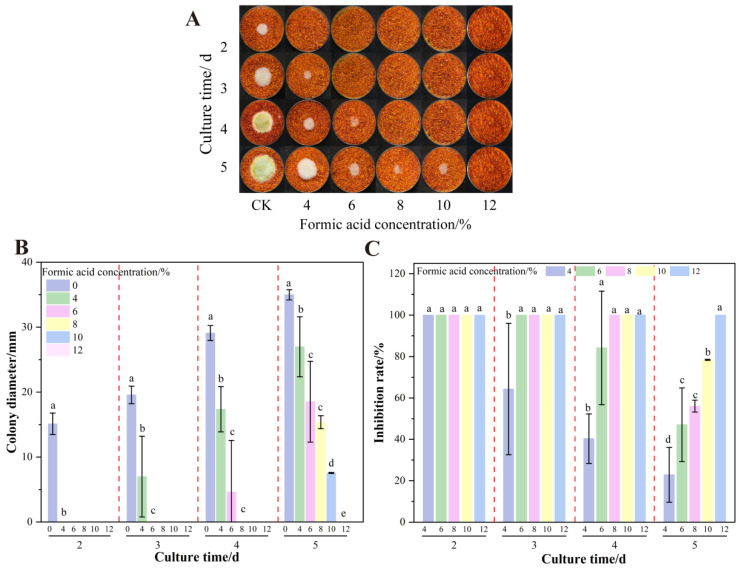
Effects of combined different formic acid concentrations on the growth of *A. flavus* on un-colonized chili powder. (**A**) Growth of *A. flavus*; (**B**) diameter of *A. flavus* colonies; (**C**) growth inhibition of *A. flavus*. Different letters at the top of the column indicate significant differences between treatment conditions (*p* < 0.05).

Using a fixed UV irradiation distance of 15 cm and total treatment duration of 60 min, varying formic acid concentrations were evaluated for their inhibitory effects on *A. flavus* in un-colonized chili powder. As shown in Figure 5, elevated formic acid concentrations significantly suppressed fungal growth, achieving 100% at 12% (*v*/*v*). On the 5th day, when the concentration of formic acid was 6%, the growth inhibition rate of colony diameter on chili powder was not significantly different from the growth inhibition rate of the 8% concentration group (*p* > 0.05). At this time, the growth inhibition rate of *A. flavus* colonies was 56.07%. To ensure effective growth suppression, concentrations ≥8% were recommended, confirming 8–12% as the optimal inhibitory range.

**Figure 6:**
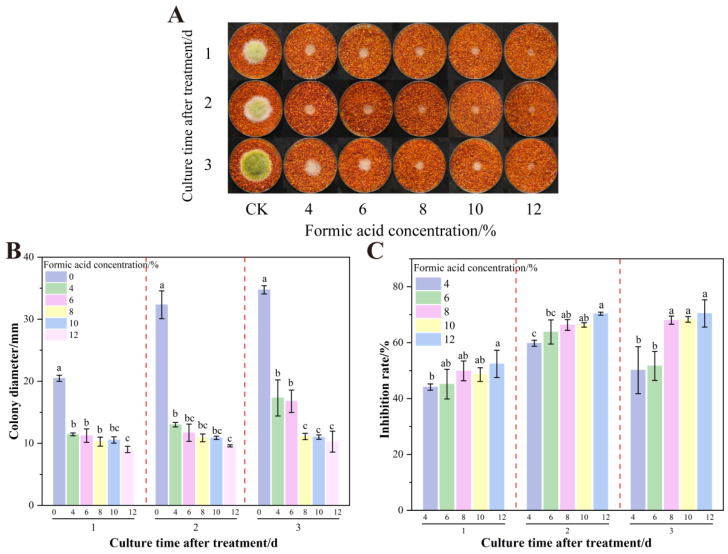
Effects of combined different formic acid concentrations on the growth of *A. flavus* on already colonized chili powder. (**A**) Growth of *A. flavus*; (**B**) diameter of *A. flavus* colonies; (**C**) growth inhibition of *A. flavus.* Different letters at the top of the column indicate significant differences between treatment conditions (*p* < 0.05).

As depicted in Figure 6, elevated formic acid concentrations progressively suppressed *A. flavus* growth on chili pepper, correlating with increased inhibition rates. Concentrations exceeding 8% significantly inhibited fungal proliferation, with near-complete growth suppression (>70% inhibition). It indicates that the concentration of formic acid treatment was selected as 8%, 10%, and 12% for the subsequent orthogonal test.

### 3.5. The Effect of UV Irradiation Distance on the Growth of A. flavus on Dried Red Chili Powder in UV-Formic Acid Treatment

**Figure 7:**
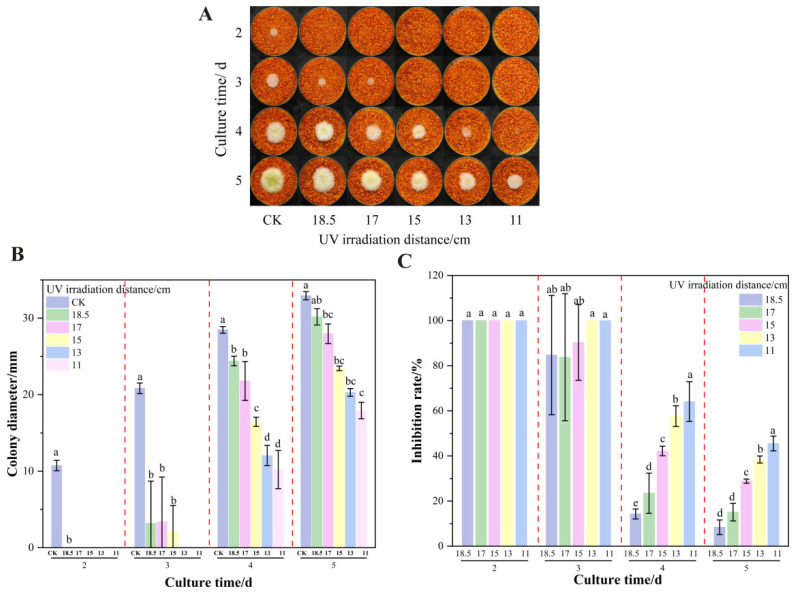
Effects of combined different UV treatment distances on the growth of *A. flavus* on un-colonized chili powder. (**A**) Growth of *A. flavus*; (**B**) diameter of *A. flavus* colonies; (**C**) growth inhibition of *A. flavus.* Different letters at the top of the column indicate significant differences between treatment conditions (*p* < 0.05).

When co-treating uncolonized chili powder with 8% formic acid and UV for 60 min under varying irradiation distances, distinct inhibitory effects on *A. flavus* growth were observed. Shortening the UV distance progressively enhanced fungal suppression, correlating with increasing colony inhibition rates (Figure 7). After 24 h of incubation, there was no significant difference in colony diameter among all treatment groups compared to the pre-treatment baselines (*p* > 0.05), indicating that the combined treatment had already achieved notable inhibitory effects. However, after 3 days of processing, significant differences were observed. The group receiving UV irradiation at 11 cm exhibited a colony diameter of 17.93 mm (45.55% inhibition rate), demonstrating potent antifungal activity. Conversely, the 18.5 cm UV group achieved only 8.4% inhibition, indicating minimal suppression. These findings collectively demonstrate that the combined use of formic acid and UV exhibited pronounced distance-dependent inhibitory effects. Shorter UV irradiation distances (corresponding to higher UV intensity) enhanced antimicrobial activity, thus effectively controlling *A. flavus* growth on chili powder.

**Figure 8:**
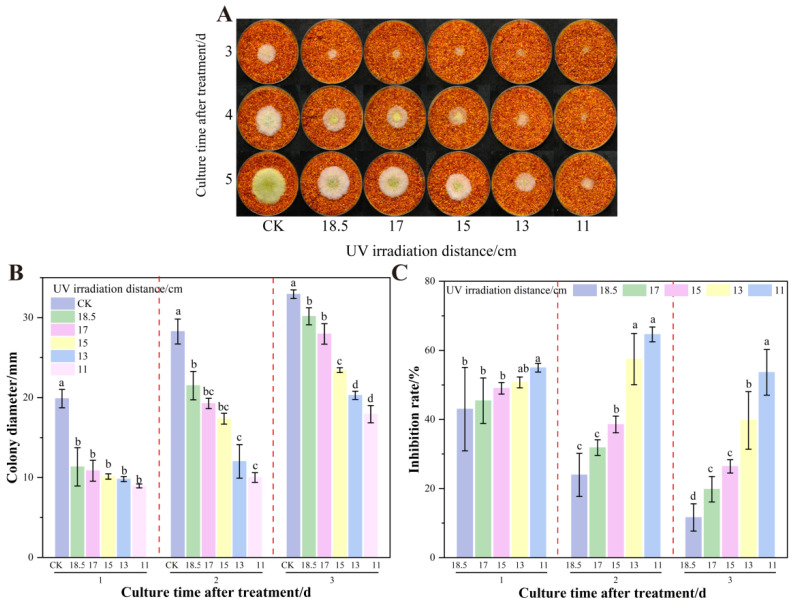
Effects of combined different UV treatment distances on the growth of *A. flavus* on already colonized chili colonies. (**A**) Growth of *A. flavus*; (**B**) diameter of *A. flavus* colonies; (**C**) growth inhibition of *A. flavus.* Different letters at the top of the column indicate significant differences between treatment conditions (*p* < 0.05).

As shown in Figure 8, the untreated control group (CK) formed completely mature colonies with mycelia spreading to the edge of the Petri dish on the 5th day of incubation. In contrast, shortening the UV irradiation distance to 11 cm significantly suppressed colony growth, and only small, localized plaques were formed (Figure 8A). Reduction from 18.5 cm to 11 cm decreased colony diameters from 30.17 mm to 17.93 mm (Figure 8B). The strongest inhibition inflection point was observed at a distance of 15 cm, when the colony diameter decreased by 26.42% compared to the CK (Figure 8C). Notably, the treatment groups showed differences on the 3rd day of processing, and the gap between the different distances widened with time, suggesting a cumulative inhibitory effect of UV treatment [51]. Based on these results, 15, 13, and 11 cm UV distances were selected for orthogonal testing.

### 3.6. The Effect of Total Treatment Time on the Growth of A. flavus on Dried Red Chili Powder in UV-Formic Acid Treatment

**Figure 9:**
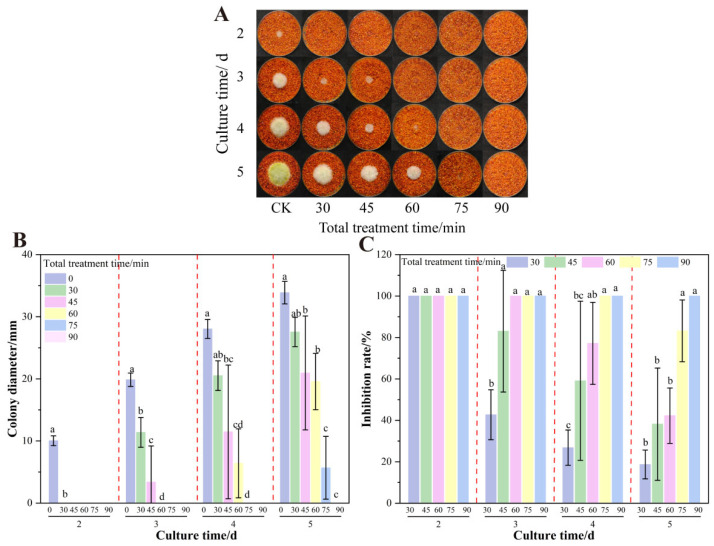
Effects of combined different treatment times on the growth of *A. flavus* on un-colonized chili powder. (**A**) Growth of *A. flavus*; (**B**) diameter of *A. flavus* colonies; (**C**) growth inhibition rate of *A. flavus.* Different letters at the top of the column indicate significant differences between treatment conditions (*p* < 0.05).

The effect of the total treatment time with formic acid and UV on the inhibitory effect was also significant. Time-dependent inhibition of *A. flavus* growth on un-colonized dried chili powder was evaluated at 30, 45, 60, 75, and 90 min intervals (Figure 9). The control group formed complete green colonies (mycelial coverage > 60%) on the 5th day of incubation, whereas the colonies in the co-treatment group gradually shrank with the prolongation of the treatment time. Particularly at ≥75 min, the colonies were almost completely stagnant, and no obvious colonies were seen to grow on chili powder (Figure 9A). In Figure 9B, the diameter of the colonies decreased from (27.55 ± 2.36) mm to 0 mm during the increase in treatment time from 30 min to 90 min on the 5th day of incubation, and the inhibition effect was gradually significant. The inhibition rate was <20% when the combined treatment time of formic acid and UV was 30 min, while the inhibition rate exceeded 80% and saturated after ≥75 min, with a high inhibition effect. Based on dose–response relationships, 60, 75, and 90 min were selected for orthogonal testing.

**Figure 10:**
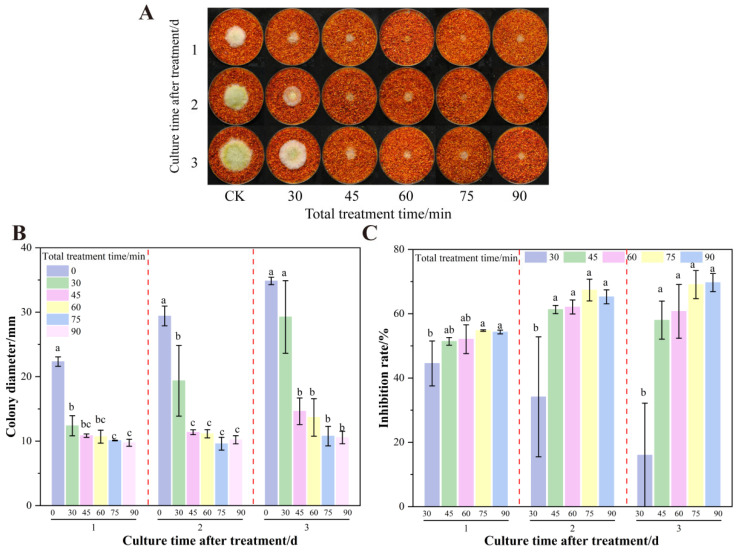
Effects of combined different treatment times on the growth of *A. flavus* on already colonized chili powder. (**A**) Growth of *A. flavus*; (**B**) diameter of *A. flavus* colonies; (**C**) growth inhibition of *A. flavus.* Different letters at the top of the column indicate significant differences between treatment conditions (*p* < 0.05).

As shown in Figure 10A, UV-formic acid co-treatment significantly altered *A. flavus* colony morphology on already colonized dried chili powder. When the treatment time was ≥45 min, the growth of colonies on chili powder basically did not grow, and the inhibition effect was obvious. Colony diameters in the treatment group were significantly lower than that of the control group (Figure 10B). The inhibition rate of colony growth could be more than 60% when the treatment time was more than 45 min (Figure 10C), which significantly inhibited the growth of *A. flavus* filaments on chili powder. At this time, with the prolongation of the treatment time, there was no significant difference in the diameter of the colonies and the inhibition rate of colony growth among the various groups (*p* > 0.05). Therefore, in combination with the above results of the inhibition on un-colonized chili powder, total treatment times of 60, 75, and 90 min were selected as the treatment levels for subsequent orthogonal tests.

### 3.7. The Effect of UV-Formic Acid Treatment Orthogonal Test on the Growth of A. flavus and AFB_1_ Synthesis on Dried Red Chili Powders

#### 3.7.1. Un-Colonized Chili Powder

**Figure 11:**
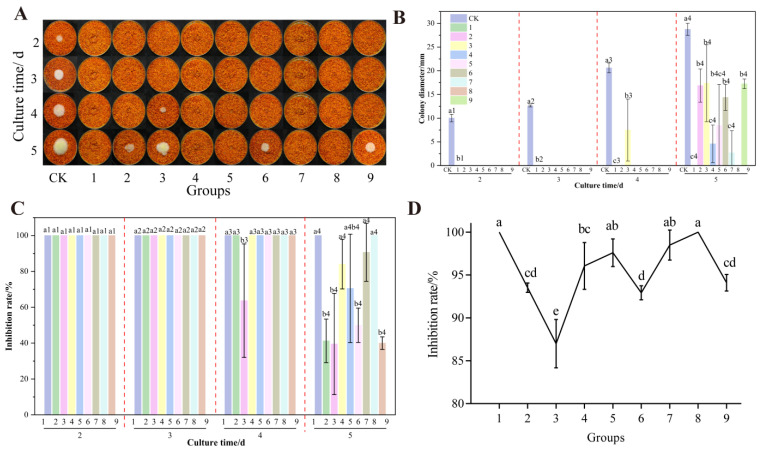
Effects of orthogonal treatments on the growth of *A. flavus* on un-colonized chili powder. (**A**) Growth of *A. flavus*; (**B**) diameter of *A. flavus*-growing colonies; (**C**) inhibition of colony growth; (**D**) inhibition of AFB_1_ synthesis. Different letters at the top of the column indicate significant differences between treatment conditions (*p* < 0.05).

The UV treatment distance (11, 13, and 15 cm), formic acid treatment concentration (8%, 10%, and 12%), and total treatment time (60, 75, and 90 min) obtained from the above results were used as orthogonal treatment factors and levels to determine the optimal treatment parameters for each of the nine sets of experiments, and the results are shown in Figure 11. Overall, these nine groups of tests showed strong inhibition of the growth and AFB_1_ synthesis of *A. flavus* on chili powder, and a better inhibitory effect was observed. After 3 days, treatment groups are not growing *A. flavus* colonies, with a growth inhibition rate of 100%. On the culture of the 5th day, the 1st group and 8th group showed the best inhibition effect on the growth of *A. flavus* colonies. The 4th, 5th, and 7th groups of colonies grown by the diameter of the colony was small and cannot be seen clearly in the picture of the mycelium growth of colonies. The growth inhibition rate was higher than 60%, and the inhibition of the production of toxicity of *A. flavus* was above 95%. Therefore, it can be concluded that after combined treatment with formic acid and UV, groups 1 (formic acid concentration of 12%, UV treatment distance of 11 cm, and treatment time of 90 min), 5 (formic acid concentration of 10%, UV treatment distance of 11 cm, and treatment time of 75 min), 7 (formic acid concentration of 8%, UV treatment distance of 13 cm, and treatment time of 90 min), and 8 (formic acid concentration of 8%, UV treatment distance of 15 cm, and treatment time of 75 min) had the optimal inhibition effect on the growth and AFB_1_ synthesis of *A. flavus* on un-colonized chili powder.

#### 3.7.2. Already Colonized Chili Powder

**Figure 12:**
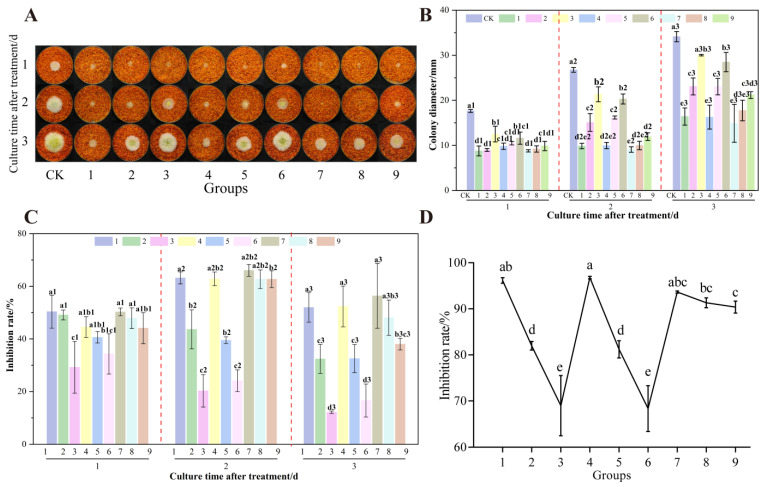
Effects of orthogonal treatments on the growth of *A. flavus* on already colonized chili powder. (**A**) Growth of *A. flavus*; (**B**) diameter of *A. flavus*-growing colonies; (**C**) inhibition of colony growth; (**D**) inhibition of AFB_1_ synthesis. Different letters at the top of the column indicate significant differences between treatment conditions (*p* < 0.05).

As shown in Figure 12, the nine groups of orthogonal treatments showed significant differences (*p* < 0.05) in the inhibition of *A. flavus* on already colonized dried chili powder. The control group (CK) colonies showed complete yellow-green morphology, while the colony morphology of the treatment group changed to different degrees with different combinations of formic acid concentration (8–12%), UV treatment distance (11–15 cm) and total treatment time (60–90 min). However, the growth of *A. flavus* on chili powder in the treatment group was significantly inhibited during the incubation process, which was significantly different from the CK in all cases (Figure 12A). On the 1st and 3rd day after processing, the colony diameters of the treated groups were generally smaller than those of the CK, showing stronger inhibition (Figure 12B). The inhibition rates of groups 1, 4 (formic acid concentration of 10%, UV treatment distance of 15 cm, and treatment time of 90 min), 7, and 8 reached more than 40%, and the inhibition rates of AFB_1_ synthesis were all greater than 90%. Overall, the combined treatment with formic acid and UV showed a synergistic efficacy on the growth and AFB_1_ synthesis inhibition of *A. flavus* on dried chili powder. Efficacy was time-dependent, aligning with observations from un-colonized chili powder.

The optimal parameter groups (groups 1, 4, 5, 7, and 8) screened by the orthogonal test showed that a lower formic acid concentration (8–12%) combined with shorter UV distance (11–15 cm) and shorter treatment time (75–90 min) could achieve high efficiency of fungal inhibition (colony inhibition > 60%, AFB_1_ synthesis inhibition >90%). Mechanistically, ≥8% formic acid concentration sufficiently compromises cell membrane integrity, potentiating UV-mediated intracellular damage. When the total treatment time was reduced to 75 min, an optimal equilibrium between formic acid volatilization and UV penetration was reached. Further prolongation of the treatment time (e.g., 90 min) may slightly increase the inhibition rate, but may increase energy consumption and induce the risk of lipid oxidation [52].

### 3.8. The Effect of UV-Formic Acid Treatment on the Quality of Dried Red Chili Powder

**Figure 13:**
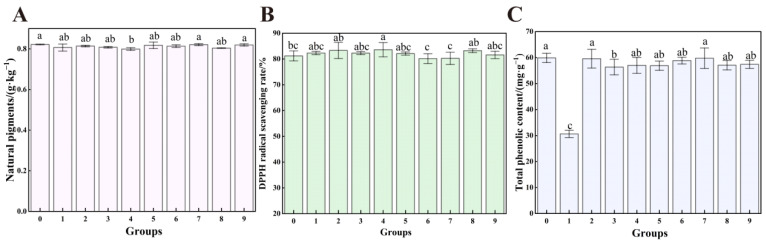
Effects of combined treatment with formic acid and UV on the quality of chili powder. (**A**) Natural coloring matter; (**B**) DPPH radical scavenging rate; (**C**) total phenol content. Different letters at the top of the column indicate significant differences between treatment conditions (*p* < 0.05).

In this study, the effect of nine groups of orthogonal test treatments on the quality of dried red chili powder was evaluated, and the results were shown in Table 3 and Figure 13. The differences in the color changes in chili powder were no significant differences, and for the brightness value *L* and the yellow-blue value *b*, the differences in the changes were not significant *(p* > 0.05), and only the red-green value *a* of chili powder in some treatment groups showed significant changes, and only the five groups of the 2nd (formic acid concentration of 12%, UV treatment distance of 13 cm, and treatment time of 75 min), 3rd (formic acid concentration of 12%, UV treatment distance of 15 cm, and treatment time of 60 min), 6th (formic acid concentration of 10%, UV treatment distance of 13 cm, and treatment time of 60 min), 8th, and 9th (formic acid concentration of 8%, UV treatment distance of 11 cm, and treatment time of 60 min). Studies have shown that pigment degradation occurs under UV treatment [53], but the pigment degradation effect of UV can be effectively attenuated by a combination of formic acid fumigation treatment, probably because organic acids have certain color-enhancing effects [54]. This synergistic interaction mitigates oxidative stress accumulation in chili powder while preserving phenolic and antioxidant activities. Figure 13A shows the content of natural coloring matter in different groups, from the results, the content of natural coloring matter in all treatment groups did not differ significantly and they were all close to the CK, indicating that the combined treatment of formic acid and UV had less of an effect on the natural coloring substances. For the spiciness of dried chili powder, after treatment, capsaicin and dihydrocapsaicin did not show significant differences between treatment groups compared to the CK (*p* > 0.05), suggesting that capsaicin in chili powder are stable in nature, are quite stable in acids and bases [55] and have high antioxidant activity [56]. They are less sensitive to UV and formic acid. This is consistent with the findings that capsaicin in chili powder did not change significantly after UV and ozone treatment of chili powder [57]. Figure 13B demonstrates the DPPH radical scavenging rates of the groups, which showed that the DPPH scavenging rates exhibited significant differences only between Group 4 and the CK, while the rest of the groups showed no significant differences compared to the CK, indicating that the combined treatment of formic acid and UV had a certain but insignificant effect on the antioxidant capacity of chili powder. Figure 13C reflects the TPC of each group, with significant differences between the TPC of groups 1, 3, and the CK, and no significant change in the TPC of the rest of the groups. It indicates that the combined treatment of formic acid and UV had some effect on the TPC of chili powder, but the overall effect was not significant. The results of some studies have also shown that UV has little effect on the polyphenol content and antioxidant activity of edible roses [58]. Combining the above results, it can be concluded that under the condition of ensuring that the quality of chili powder is not significantly affected, the treatment that has a significant effect on the inhibition of the growth of *A. flavus* is the 8th group. The optimal treatment was determined as formic acid concentration of 8%, UV treatment distance of 15 cm, and treatment time of 75 min as the optimal way of dealing with the growth of *A. flavus* in the dry red chili powder. The optimal treatment of 8% formic acid, a UV treatment distance of 15 cm, and a treatment time of 75 min was determined to be the best way to treat the growth of *A. flavus* in dried red chili powder, which not only significantly inhibited the growth and toxigenicity of *A. flavus* but also reduced the impact on the quality of chili powder. However, formic acid residues (even trace amounts) may catalyze Maillard reactions in hot and humid environments, leading to accelerated browning [52]. Therefore, fumigation of chili powder using this method should be followed by ventilation to allow the rapid evaporation of residual formic acid gas from the chili powder and avoid quality changes in the later stages of storage.

### 3.9. UV-Formic Acid Treatment Induces Cell Membrane Alterations in A. flavus

**Figure 14:**
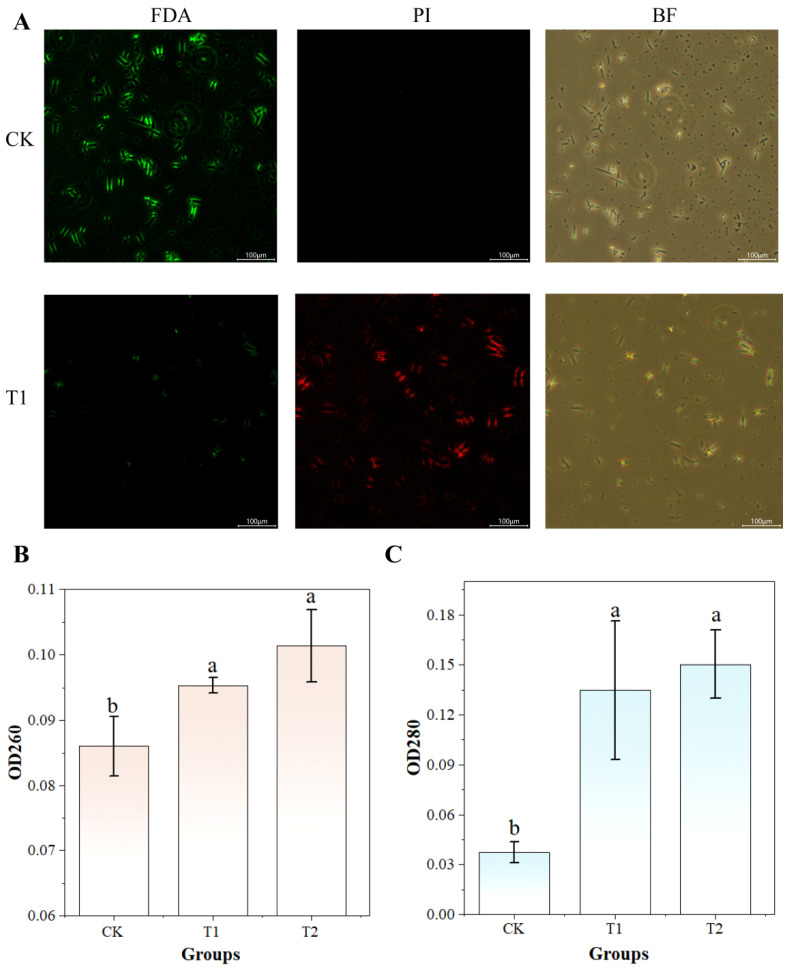
The effect of UV-formic acid treatment on the membrane integrity of *A. flavus* cells. (**A**) Fluorescence effect of FDA and PI staining; (**B**) nucleic acid leakage; (**C**) protein leakage. Different letters at the top of the column indicate significant differences between treatment conditions (*p* < 0.05).

**Figure A1:**
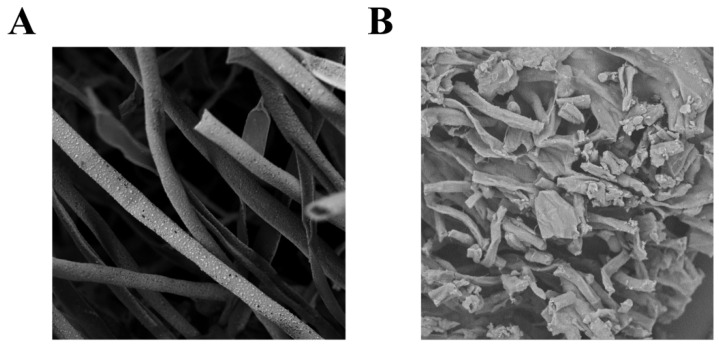
The effect of UV formic acid combined treatment on the morphological structure of *A. flavus*. (**A**) Untreated group; (**B**) T1-treated group.

To evaluate UV-formic acid-induced damage to *A. flavus* cell membranes, membrane integrity was assessed under two treatments (T1, T2) using FDA/PI fluorescence microscopy. The absorbance at 260 nm and 280 nm was also measured to characterize the amount of leaked nucleic acids and proteins in the cells. The content of leaked nucleic acids and proteins increased once the cell membrane was damaged [39]. As shown in Figure 14A, the *A. flavus* spore suspension from group T1 showed intense red fluorescence (PI staining) and diminished green signal (FDA). In contrast, the *A. flavus* spore suspension of the control group showed strong green fluorescence with minimal red staining. This indicates that the combined UV-formic acid treatment damaged the cell membrane integrity of *A. flavus*. As shown in Figure 14B,C, both OD_260_ and OD_280_ values increased following UV-formic acid treatment compared to controls. This indicates enhanced leakage of intracellular nucleic acids and proteins, with no significant difference between T1 and T2 groups (*p* > 0.05). This may be due to the fact that the cell membrane had been damaged more severely in the T1 treatment, so the content of nucleic acids and proteins leaked from the cell membrane did not increase significantly under the higher level of treatment. Also, Figure A1 confirms morphological disruption in T1-treated *A. flavus*, exhibiting membrane folds and fractures. Thus, UV-formic acid co-treatment can disrupt the cell membrane integrity of *A. flavus*. It leads to leakage of intracellular material. This is consistent with the findings obtained by Xu et al. [59]. Combined UV and peroxyacetic acid treatment can cause damage to the cell membrane, leading to leakage of intracellular substances.

### 3.10. UV-Formic Acid Induced Antioxidant Response and Energy Metabolic Reprogramming in A. flavus

**Figure 15:**
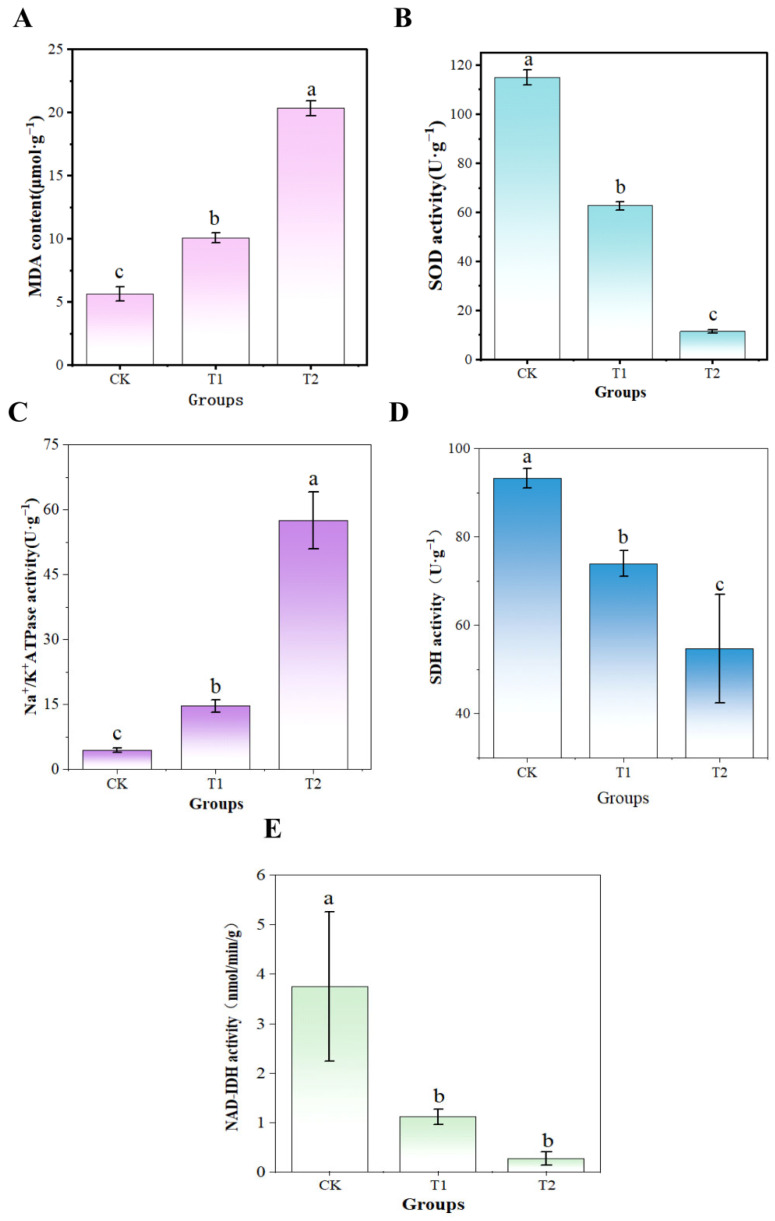
Effect of UV-formic acid treatment on *A. flavus.* (**A**) MDA content; (**B**) SOD activity; (**C**) Na^+^/K^+^-ATPase activity; (**D**) SDH activity; (**E**) NAD-IDH activity. Different letters at the top of the column indicate significant differences between treatment conditions (*p* < 0.05).

When microorganisms encounter abiotic stresses, they activate endogenous antioxidant systems to scavenge superoxide anions and free radicals, enhancing cellular defense [60]. MDA, a terminal product of lipid peroxidation, directly reflects oxidative damage to membrane lipids. Excessive accumulation of reactive oxygen species (ROS) generating oxidative stress may result in damage to biomolecules such as lipids and proteins, leading to lipid peroxidation [61]. To assess the oxidative stress induced by UV-formic acid treatment in *A. flavus*, MDA content and superoxide dismutase (SOD) activity were measured. As shown in Figure 15, the MDA content in the T1 and T2 groups after UV-formic acid treatment increased gradually with the deepening of the treatment. The SOD activity decreased gradually. Compared with the CK, both showed significant changes (*p* < 0.05). It indicates that the lipid peroxidation level of *A. flavus* mycelium increased and the antioxidant capacity was weakened after UV-formic acid treatment. Mycelial oxidative stress was enhanced, leading to irreparable oxidative damage to the structure of *A. flavus*.

Na^+^/K^+^-ATPases are enzymes present in almost all cytoplasmic membranes. Converting the energy of ATP into transmembrane Na^+^ and K^+^ gradients consumes a large portion of ATP in cells and tissues [62]. Mitochondria are key organelles in the cells of higher organisms that can produce ATP through energy metabolic pathways. This is necessary for cellular physiological activity [63]. Mitochondrial dehydrogenases contain a variety of dehydrogenases in mitochondria that are key catalyzing enzymes for ATP biosynthesis, such as SDH and NAD-IDH [64]. SDH catalyzes the conversion of succinate to fumarate and facilitates ATP production. It has a large role in the TCA cycle and electron transport chain [65]. Therefore, the inhibitory mechanism of growth inhibition of *A. flavus* by UV-formic acid treatment was further investigated by determining the activities of energy metabolism-related enzymes SDH and NAD-IDH. As shown in Figure 15C, the Na^+^/K^+^-ATPase activity of *A. flavus* was significantly elevated after UV-formic acid treatment. This may be due to the fact that the UV-formic acid treatment induced a stress response in the cells, leading to an increase in ATPase activity, which affected the energy metabolism of *A. flavus*. The results shown in Figure 15D,E informed that both SDH and NAD-IDH enzyme activities decreased after UV-formic acid treatment, with significant decreases compared to the control group. The results indicate that UV-formic acid treatment leads to a decrease in mitochondrial dehydrogenase activities of *A. flavus* filaments. These decreased enzyme activities disrupt energy metabolism in *A. flavus* cells. This leads to the occurrence of mitochondrial dysfunction in *A. flavus* mycelium, which prevents *A. flavus* from normal growth and toxicity production.

## 4. Conclusions

This study investigated UV-formic acid co-treatment efficacy against *A. flavus* in dried red chili powder. The results indicated that the combined treatment of formic acid and UV had significant advantages over the single treatments, achieving >60% growth inhibition and >90% aflatoxin B_1_ (AFB_1_) suppression within 90 min. This strategy shortens the treatment cycle to 75 min compared with single formic acid fumigation for 24 h and effectively reduces the effect of formic acid residual odor on the quality of chili powder. The synergistic effect of the two treatments was verified and the best combined effect was achieved by the simultaneous application of formic acid and UV irradiation. Through orthogonal tests and evaluation of the quality of dried red chili powder, it was determined that the optimal parameter group (formic acid concentration at 8%, UV distance of 15 cm, and treatment duration of 75 min) not only efficiently inhibited the growth and toxicity of *A. flavus*, but also did not have a significant effect on the quality indexes of chili powder, such as color and capsaicin (*p* > 0.05). The mechanism exploration results demonstrate that the combined action of UV-formic acid treatment can disrupt the cell membrane structure of *A. flavus*. They can disrupt antioxidant and energy metabolism systems, leading to mitochondrial dysfunction. Therefore, this study confirms that this technology has the dual advantages of efficient fungal inhibition and quality protection. Limitations of this study include its primary focus on *A. flavus*; the efficacy against other mycotoxigenic fungi (e.g., *A. parasiticus*, *A. ochraceus*) or common spoilage organisms relevant to spices remains unexplored. Furthermore, the findings are derived from a single food matrix (dried red chili powder) with a limited sample size, necessitating validation for other spice powders or complex food systems. The absence of post-treatment storage stability data is another limitation, and the practical gap between controlled laboratory settings and industrial-scale application needs consideration. Regarding practical implementation, while the costs of formic acid and UV-C technology are relatively low and energy consumption manageable, several challenges warrant attention. Scaling up this treatment would require specialized equipment for the safe generation and containment of formic acid vapor combined with uniform UV-C irradiation across large volumes of powder, posing significant engineering hurdles. Ensuring worker safety regarding formic acid exposure and UV radiation necessitates stringent protocols. Crucially, regulatory approval pathways are essential; establishing a safety profile according to food regulations is a critical prerequisite for commercial adoption. Moreover, costs are relatively low, and energy consumption is manageable. The treatment parameters can be adjusted according to the actual situation, and the operation is relatively simple, which has certain practical application significance. It can provide a feasible solution for the prevention and control of *A. flavus* contamination in the production of dried red chili powder. This study is limited to a single food substrate (with a small sample size) and lacks experimental investigation of the storage period after treatment. In the future, it could be expanded to complex food systems and in-depth research on mechanisms of action can be conducted.

## Figures and Tables

**Table 1:** Design of the UV-formic acid combination approach.

Combined Approach	Groups	Time Distribution/min
CK *	W1	0
Single	W2	90 UV
	W3	90 FA
Link together	W4	45 FA + 45 UV
	W5	45 UV + 45 FA
First single, then parallel	W6	45 FA + 45 FA/UV
	W7	45 UV + 45 FA/UV
First parallel, then single	W8	45 FA/UV + 45 FA
	W9	45 FA/UV + 45 UV
Parallel	W10	90 FA/UV

* CK indicates chili powder untreated. FA represents formic acid.

**Table 2:** Orthogonal experiment factors and levels design table.

Groups	Treatment		
	Formic Acid Concentration/%	Total Treatment Time/min	UV Distance/cm
1	12	90	11
2	12	75	13
3	12	60	15
4	10	90	15
5	10	75	11
6	10	60	13
7	8	90	13
8	8	75	15
9	8	60	11

**Table 3:** Effect of UV synergistic formic acid treatment on the quality of dried red chili powder. (The brightness (*L*), red–green (*a*), and yellow–blue (*b*) values).

Index	CK	Groups								
		1	2	3	4	5	6	7	8	9
*L*	48.336 ± 0.890 a	47.473 ± 1.194 a	48.326 ± 0.649 a	48.273 ± 1.063 a	47.879 ± 0.479 a	47.386 ± 0.603 a	47.659 ± 0.362 a	47.691 ± 0.421 a	48.061 ± 0.260 a	47.919 ± 0.163 a
*a*	19.467 ± 0.208 a	16.867 ± 0.513 d	18.533 ± 1.365 abc	18.600 ± 0.436 abc	17.867 ± 0.252 bcd	16.567 ± 0.379 d	18.767 ± 0.802 ab	17.633 ± 0.289 cd	18.333 ± 0.153 abc	18.767 ± 0.058 ab
*b*	21.302 ± 0.795 a	20.034 ± 1.726 a	20.438 ± 1.313 a	21.077 ± 1.091 a	20.176 ± 0.968 a	20.806 ± 1.219 a	20.608 ± 0.945 a	20.308 ± 0.937 a	20.315 ± 1.292 a	20.541 ± 0.980 a
Capsaicin/(g·kg^−1^)	0.944 ± 0.039 a	0.998 ± 0.089 a	0.966 ± 0.007 a	0.958 ± 0.044 a	0.944 ± 0.045 a	0.972 ± 0.006 a	0.982 ± 0.008 a	0.947 ± 0.033 a	0.966 ± 0.045 a	0.939 ± 0.055 a
Dihydrocapsaicin/(g·kg^−1^)	0.362 ± 0.018 a	0.384 ± 0.031 a	0.364 ± 0.005 a	0.360 ± 0.012 a	0.353 ± 0.012 a	0.365 ± 0.003 a	0.366 ± 0.005 a	0.354 ± 0.010 a	0.364 ± 0.017 a	0.354 ± 0.018 a
Total capsaicin/(g·kg^−1^)	1.451 ± 0.064 a	1.535 ± 0.134 a	1.478 ± 0.014 a	1.463 ± 0.063 a	1.441 ± 0.064 a	1.485 ± 0.010 a	1.498 ± 0.014 a	1.446 ± 0.047 a	1.478 ± 0.070 a	1.437 ± 0.081 a

The values are means ± standard deviation (n = 3). Different letters in the same row and section indicate that values are significantly different (*p* < 0.05). CK represents the untreated group.

## Data Availability

The data will be made available upon request from the corresponding author. The data are not publicly available due to privacy restrictions.

## Abbreviations

The following abbreviations are used in this manuscript:

UV	Ultraviolet
AFB_1_	Aflatoxin B_1_
TPC	Total phenolic content
DPPH	2,2-Diphenyl-1-picrylhydrazyl
FDA	Fluorescein diethyl acetate
PI	Propidium iodide
SEM	Scanning electron microscopy
MDA	Malondialdehyde
NAD-IDH	NAD-isocitrate dehydrogenase
SDH	Succinate dehydrogenase
